# The relationship between substance use disorder and gambling disorder: A nationwide longitudinal health registry study

**DOI:** 10.1177/14034948211042249

**Published:** 2021-09-30

**Authors:** Tony Leino, Torbjørn Torsheim, Mark D. Griffiths, Ståle Pallesen

**Affiliations:** 1 Department of Psychosocial Science, University of Bergen, Norway; 2 International Gaming Research Unit, Psychology Department, Nottingham Trent University, UK; 3 Norwegian Competence Center for Sleep Disorders, University of Bergen, Norway; 4 Optentia Research Focus Area, North-West University, Vanderbijlpark Campus, South Africa

**Keywords:** Gambling disorder, substance use disorders, comorbidity, population health registry data

## Abstract

*Aim:* This study aimed to examine the co-morbidity and temporal relationship between substance abuse disorders (SUDs) and gambling disorder (GD). *Method:* Cross-tabulated census data were retrieved from the Norwegian Patient Registry. The data included the number of patients by year of first-time incidence of GD and/or SUD diagnoses, age and sex from 2008 to 2017. *Results:* Approximately 22.5% of GD patients were also diagnosed with SUD, whereas 0.7% of SUD patients were also diagnosed with GD. Among GD patients, males had a greater risk of SUD in the same year compared to females, whereas the risk of SUD a year or more after the onset of GD was greater among females compared to males. Among SUD patients, males had a greater risk of GD in all age categories and across all time periods except among those aged 40–66 years. The risk of GD three to four years after the onset of SUD among those aged 40–66 years was similar between SUD males and females. *Discussion:* The overall co-morbidity of SUD and GD was low. However, the risk of the other addictive disorder was contingent upon the nature of the first disorder. The risk of SUD among GDs over time was greater among females compared to males. ***Conclusions:* The risk of the other addictive disorder appears to be contingent upon the first addictive disorder. There are sex differences in the risk trajectories of the other addictive disorder over time between GD patients and SUD patients**.

## Introduction

Gambling disorder (GD) is a persistent and recurring maladaptive gambling behaviour affecting personal, family and occupational pursuits [1]. To be diagnosed with GD based on the criteria found in the (current) fifth edition of the *Diagnostic and Statistical Manual for Mental Disorders* (DSM-5), an individual must experience at least four out of nine symptoms within a 12-month period [1]. GD and pathological gambling (PG) are often used synonymously due to similar clinical criteria of clinical gambling diagnosis. In contrast, gambling problems implicates negative consequences of gambling without necessarily fulfilment of the diagnostic criteria of a clinical GD diagnosis [2]. The prevalence of GD is approximately 1% in the general adult population (aged ⩾18 years) [3,4], whereas almost 8% have experienced significant gambling-related problems during their lifetime [5]. Norwegian nationally representative gambling studies show that the proportion of problem gamblers has increased from 0.6% in 2013 to 0.9% in 2015 and 1.4% in 2019 [6,7], with the increase from 2015 to 2019 being statistically significant.

Substance use disorders (SUDs) are mental and behavioural disorders reflecting misuse of psychoactive substances [1]. Such disorders include excessive use of alcohol and/or different drugs such as nicotine and other psychoactive substances, respectively (see codes F10–F19 in the 10th revision of the *International Classification of Diseases* (ICD-10) manual for a complete list) [8]. Problematic alcohol use is the most common type of SUD in Norway [9], with 7% reporting having an alcohol use disorder (AUD) in 2016 [10]. Cannabis is the most common illicit drug type in Norway [9], with approximately 4% of the adults (aged 16–64 years) reporting such misuse in the past 12 months in the years 2014–2016 [11]. Recognising commonalities in core criteria and symptoms (e.g. tolerance and impaired control), co-morbidity, genetic make-up, neurobiology and treatment approaches between GD and SUD [12] has led to the inclusion of GD as the first non-chemical (i.e. behavioural) addiction in the DSM-5 [1].

The co-occurrence between SUD and GD is well recognised [3,13] and has been empirically demonstrated in comprehensive reviews (e.g. Sussman et al. [14]). A higher proportion of individuals with GD suffer from SUD compared to the general population [12]. For example, Welte et al. found that 24% of those with GD had AUD, whereas 1.4% of non-GDs met the criteria for AUD [15]. A meta-analysis of population-based surveys reported that 60% of those with GD were nicotine dependent, while 58% suffered from other SUDs [16]. Still, an unpublished reanalysis of the Norwegian national gambling survey [7] indicates minor differences in risky drinking patterns between problems gamblers and non-gamblers, where approximately 35% of the problem gamblers had a risky drinking pattern compared to 31% of the non-gamblers. The prevalence of SUD is high among treatment-seeking individuals with GD. A Norwegian study reported that 75% of in-treatment pathological gamblers had misused alcohol, whereas 59% of them had harmful and hazardous drinking [17]. A recent Swedish nationwide register study between the years 2005 and 2016 found that approximately 25% of individuals with GD had a co-occurring substance-related disorder [18].

Similarly, GD appears to be prevalent among those with mild to moderate alcohol dependence [19]. Among a clinical subsample of 140 outpatient alcohol addicts, 12% and 10% met the criteria for problem gambling and PG, respectively [20]. Another study reported that approximately 10% of patients seeking treatment for SUD suffered from GD [21]. In line with these findings, 11% of individuals seeking treatment for AUD/drug use disorder (DUD) reported gambling problems at the time of treatment, while 15% had suffered from GD during their lifetime. A meta-analysis found that approximately 14% of SUD patients experienced GD [22]. However, a nationally representative US sample found a significantly lower prevalence of PG among those with AUD (1.0%), DUD (1.6%) and nicotine dependence (1.5%) [4]. The unpublished reanalysis of the Norwegian gambling survey [7] (see above) showed that approximately 1.3% of those with a risky drinking pattern were problem gamblers, suggesting a discrepancy in prevalence rates between nationally representative samples and treatment-seeking samples.

Despite the relatively large amount of research on the co-occurrence of GD and SUD, few studies have examined the temporal relationship between GD and SUD. In one study, it was found that among individuals with a history of both SUDs and GD, 36.2% had an earlier onset of PG, 57.4% had an earlier onset of any SUD, while 6.4% experienced PG and SUD in the same year [2]. This suggests that most often SUDs precede PG. However, there is a paucity of studies examining the prospective relationship between different addictive disorders. In addition, to the best of the authors’ knowledge, no study has examined demographic factors associated with the prospective relationships between such disorders.

Overall, the findings show that SUD is more prevalent among GD sufferers than vice versa. Still, the vast majority of studies investigating the relationship between SUD and GD are based on clinical and non-randomised samples (e.g. Bu and Skutle [17], Sellman [19], Grant et al. [23], Leavens et al. [24] and Maccallum and Blaszczynski [25]) where two studies have used health registry data [17] or nationally representative samples [4]. Consequently, the aim of the present study was to describe the relationship between GD and SUD by conducting a prospective study using health registry data. First, we examined the co-morbidity between SUD and GD, irrespective of onset of the two addictive disorders. Second, we examined the time lag and relative risk of developing the other addictive disorder among patients with an existing addictive disorder by sex within the same age cohorts in a four-year period.

## Methods

Nationwide Norwegian health register data from the Norwegian Patient Registry (NPR) were retrieved. The NPR was established in 1997 by the private Norwegian research institute SINTEF, and it transferred to the Norwegian Directorate of Health in 2007. Since 2008, the register has contained a personal identification number (PIN), as well as medical information such as hospital stays, diagnoses according to the ICD-10 and inpatient and outpatient treatment provided by public regional health authorities or specialised treatment providers on behalf of all public regional health authorities. Data from mental health-care facilities for adults were included in 2001. From 2006 to 2010, the NPR also included information on specialised interdisciplinary addiction treatment [26]. Cross-tabulated census data of those who received a GD and/or SUD diagnosis in the years from 2008 to 2017 were received from the NPR. The frequency of patients was categorised by age (⩽17 years, 18–39 years, 40–66 years and ⩾67 years), sex, year of first registered GD diagnosis (code F63.0 according to the ICD-10) and/or year of first registered SUD diagnosis (codes F10–F19 according to ICD-10) in the NPR since 2008. Data from those aged ⩽17 years (*n*=5,178) were excluded from the data set due to the high probability that those in this age category actually suffered from videogame addiction (rather than GD) and that the F63.0 diagnosis was set since gaming addiction came ‘closest’ to GD.

### Analyses

The analyses comprised two parts. The first analysis examined the co-morbidity of GD and SUD, irrespective of relative onset of the two addictive disorders. In the preceding analysis, only those years where GD(SUD) could be followed for four complete years after the onset of SUD(GD) were included (i.e. onset of SUD(GD) ranged from 2008 to 2013, whereas years of co-morbid GD(SUD) ranged from 2008 to 2017). The temporal lag between the first and the other addictive disorder was then analysed among patients with GD or SUD. The temporal difference between the first addictive disorder and the other addictive disorder was categorised as (i) patients who were diagnosed with both addictive disorders in the same year, (ii) patients who were diagnosed with the other addictive disorder one to two years after the onset of the first addictive disorder and (iii) patients who were diagnosed with the other addictive disorder three to four years after the onset of the first addictive disorder. The analyses were stratified by age and sex. Age was categorised as 18–39 years, 40–66 years and ⩾67 years.

Data management was carried out utilising R v3.5.1 (The R Foundation for Statistical Computing, Vienna, Austria). *Risk* and *relative risks* were calculated using Microsoft Excel. No inference-based statistics were used because the data included the whole population of patients with an addictive disorder and year of first-time incidence of GD and/or SUD from 2008 to 2017.

### Ethics

Because the data comprised a frequency table of GD and/or SUD patients by year of diagnosis, age and sex, the data were regarded as anonymous and thus exempted from approval from the Regional Committee for Medical and Health Research Ethics. Consequently, approval of the study was solely provided by the NPR.

## Results

### Descriptive statistics

Table 1 shows demographic statistics for different exposure groups with an addictive disorder pooled over time (from 2008 to 2017). A total of 151,144 patients received their first addictive disorder (SUD and/or GD) diagnosis between 2008 and 2017. There were 4,388 GD patients (GDs; 19% females), corresponding to 2.9% of all patients with addictive disorders. Approximately 63.9% were aged 18–39 years, 35.3% were aged 40–66 years, whereas 0.8% where aged ⩾67 years. Approximately 81% of the GDs were males aged between 18 and 66 years. Among female GD patients, the incidence of GD was relatively stable from 18–39 years of age to 40–66 years of age but became steeply reduced among those aged ⩾67 years. Among male GD patients, the incidence of GD was clearly negatively related to age. There were 147,743 SUD patients (34% female), which corresponded to 97.7% of all patients with an addictive disorder. Among SUD patients, approximately 48.0% were aged 18–39 years, 42.3% were aged 40–66 years old, whereas 9.7% were aged ⩾67 years, and 34.4% were female. Approximately 60% of all SUD patients were males aged between 18 and 66 years. The incidence of SUD declined between those aged 18–39 years and those aged 40–66 years for both sexes. A total of 987 (0.7%) patients were diagnosed with both addictive disorders, irrespective of the relative temporal onset of GD and SUD. An approximately similar demographic distribution was found among those with both disorders and those with GD. Furthermore, 22.5% of the GD patients were diagnosed with SUD, whereas 0.7% of the SUD patients were diagnosed GD.

**Table 1.** First-time incidence of GD and/or SUD across different exposure groups by age and sex.

	18–39 years				40–66 years				⩾67 years			
	Females		Males		Females		Males		Females		Males	
	*n*	%	*n*	%	*n*	%	*n*	%	*n*	%	*n*	%
GD or SUD	24,582	16.3%	48,440	32.0%	21,418	14.2%	42,337	28.0%	5,497	3.6%	8,870	5.9%
GDs	372	8.5%	2,430	55.4%	412	9.4%	1,138	25.9%	8	0.2%	28	0.6%
SUDs	24,299	16.4%	46,563	31.5%	21,089	14.3%	41,453	28.1%	5,490	3.7%	8,849	6.0%
GD and SUD	89	9.0%	553	56.0%	83	8.4%	254	25.7%	*	*	7	0.7%

* *n*⩽5 are not reported.

GD: gambling disorder; SUD: substance use disorder; GD or SUD: first-time incidence of GD or SUD in the time period from 2008 to 2017; GDs: first-time incidence of patients with GD (F63.0 according to the ICD-10); SUDs: first-time incidence of patients with SUD (F10–F19 according to the ICD-10). GD and SUD: incidence of patients with GD and SUD regardless of the relative onset of the disorders.

### The relative risk of the other addictive disorder among GDs and SUDs over a four-year period

Table 2 shows the descriptive statistics of the risk of the other addictive disorder over a four-year period among patients with GD and SUD across sex and age. Only data covering years where the other addictive disorder could be followed for four complete years after the first addictive disorder was diagnosed were included (i.e. onset of the first addictive disorder ranged from 2008 to 2013, whereas years of the other addictive disorder ranged from 2008 to 2017). Removing data from 2014 onwards resulted in the removal of 2,089 patients with GD. Furthermore, years where first-time SUD preceded first-time GD resulted in the removal of a further 138 patients with GD. The final GD population comprised 2,161 patients. Similarly, removing data covering the years 2014 onwards resulted in the removal of 45,906 SUD patients. Furthermore, removing data from years where first-time GD preceded first-time SUD resulted in the removal of 94 additional SUD patients. The final SUD population comprised 101,743 SUDs.

**Table 2.** Descriptive statistics and risk of the other addictive disorder among GD and SUD patients by age and sex.

Sex	Age	SUD|GD_N_				R_SUD|GD_			GD|SUD_N_				R_GD|SUD_		
		GD_*n*_	SUD_Same-year_	SUD_1–2yrs_	SUD_3–4yrs_	SUD_Same-year_	SUD_1–2yrs_	SUD_3–4yrs_	SUD_*n*_	GD_Same-year_	GD_1–2yrs_	GD_3–4yrs_	GD_Same-year_	GD_1–2yrs_	GD_3–4yrs_
Female	18–39	181	20	7	7	11.0%	3.9%	3.9%	16,432	20	10	9	0.12%	0.06%	0.05%
	40–66	206	17	7	7	8.3%	3.4%	3.4%	14,884	17	9	9	0.11%	0.06%	0.06%
	67+	7	*	0	0	*	0.0%	0.0%	3,103	*	0	0	*	0.00%	0.00%
Male	18–39	1,169	130	44	27	11.1%	3.8%	2.3%	31,982	130	53	44	0.41%	0.17%	0.14%
	40–66	590	72	12	15	12.2%	2.0%	2.5%	30,075	72	32	18	0.24%	0.11%	0.06%
	67+	8	*	0	0	*	0.0%	0.0%	5,267	*	0	0	*	0.00%	0.00%
Overall		2,161	243	70	56	11.2%	3.2%	2.6%	101,743	243	104	80	0.24%	0.10%	0.08%

* *n*⩽5 are not reported.

SUD|GD: SUD among GD patients; GD|SUD: GD among SUD patients; R: risk; Same-year: got GD and SUD the same year; 1–2yrs: got SUD(GD) one to two years after the onset of GD(SUD); 3–4yrs: got SUD(GD) three to four years after the onset of GD(SUD); Overall: risk of the other addictive disorder across age and sex.

Table 2 shows descriptive statistics of the risk of the other addictive disorder among GD and SUD patients, broken down by age and sex. Among GD patients, 17.1% were diagnosed with SUD in a four-year period after the onset of GD. Most GD patients were diagnosed with their first-time SUD in the same year as their GD. The results overall show that 11.2%, 3.2% and 2.6% got SUD in the same year, one to two years and three to four years after the onset of GD, respectively. Some age and sex differences were noted. A greater proportion of males were diagnosed with SUD in the same year as GD compared to females, whereas a greater proportion of females were diagnosed with SUD a year or more after the onset of GD compared to males. The number of GD patients was very small among those aged ⩾67 years, and all who got SUD did so in the same year as the GD diagnosis was made. Similarly, among SUD patients, 0.42% were diagnosed with GD in a four-year period after the onset of SUD. The overall statistics show that 0.24%, 0.10% and 0.08% were diagnosed with GD in the same year, one to two years and three to four years after the onset of SUD, respectively. A greater proportion of males than females were diagnosed with GD across all age and time categories, except among SUD patients aged 40–66 years where the proportion of GD between males and females were similar three to four years after SUD.

Table 3 shows the effect of sex in the development of the other addictive disorder among GD and SUD patients in different age cohorts. Among GD patients, males had a greater risk of SUD in the same year compared to females, except among those aged 18–39 years. The risk of SUD in the same year as GD among those aged 18–39 years was similar between males and females. Furthermore, the risk of SUD a year or more the after the onset of GD was greater among females compared to males, except among those aged 18–39 years. The risk of SUD one to two years after the onset of GD among those aged 18–39 years was similar between males and females. Among SUD patients, males had a greater risk of GD in all age categories and across all time periods, except among those aged 40–66 years. The risk of GD three to four years after the onset of SUD among those aged 40–66 years was similar between males and females.

**Table 3.** Effect of sex in the development of the other addictive disorder among GD and SUD patients by age cohort.

	RR_SUD|GD_			RR_GD|SUD_		
	SUD_Same-year_	SUD_1–2yrs_	SUD_3–4yrs_	GD_Same-year_	GD_1–2yrs_	GD_3–4yrs_
F18–39 vs. M18–39 (ref.)	0.99	1.03	1.67	0.30	0.37	0.40
F40–66 vs. M40–66 (ref.)	0.68	1.67	1.34	0.48	0.57	1.01
F67+ vs. M67+ (ref.)	0.38	–	–	0.57	–	–

F: female; M: male. RR_SUD|GD_: relative risk of SUD among GDs across sex: RR_GD|SUD_: relative risk of GD among SUDs across sex.

## Discussion

The aim of the present study was to examine the relationship between GD and SUD using health register census data from the NPR. There was a very small co-morbidity between SUD and GD among patients who received treatment by regional health authorities or specialised treatment providers on behalf of public regional health authorities in Norway because 0.7% of the population of patients with at least one addictive disorder developed both. However, the results clearly showed that the risk of another addictive disorder depended upon the nature of the first. Just under a quarter of the GD patients were diagnosed with SUD (22.5%), which is in line with previous findings [4,17–19] and findings from Sweden using corresponding methodology [18]. Only 0.7% of SUD patients were diagnosed with GD, showing that GD is not common among SUD patients. This is consistent with survey-based findings [4] and an unpublished reanalysis by the present authors of a nationally representative sample (see Introduction), albeit inconsistent with findings from non-representative clinical studies [22]. The discrepancy between these two sets of study designs may reflect methodological issues. For example, Berkson’s bias occurs when a sample is taken from a subpopulation rather than the general population. The high prevalence of GD among SUD patients found in non-representative clinical studies may overestimate the actual prevalence of SUD among GD patients in society. Another explanation for the discrepancy in results across the two sets of study designs is that treatment-seeking SUD patients found in non-representative clinical studies are more likely to suffer from GD. Given that this study was based on longitudinal nationwide population data with findings approximately similar to results from nationally representative studies, it seems conceivable that the results here are representative for the study population. Still, it can be argued that the present study underestimates the actual number of GD patients in the Norway. For example, the present study population only included individuals who had received a diagnosis and treatment in a hospital or other specialised treatment facilities, and it excluded those who experienced gambling problems but did not receive or seek treatment for their disorder(s). Findings show that there is a large gap between those treated for GD and those reporting that they suffer from gambling problems. For example, the first-time incidence in this study included 4,388 GD patients in a 10-year period, whereas approximately 55,000 individuals were reported to suffer from problem gambling in 2019 [7]. In line with previous findings, this suggests that only a minority of those with gambling problems seek formal gambling treatment where natural recovery is common among individuals with gambling problems [27]. Indeed, approximately 80% of problem gamblers who manage to stop gambling do so without any formal help [28]. However, it should still be noted that gambling severity and negative consequences are higher among those problem gamblers who access treatment compared to those who recover spontaneously without treatment [28].

The second analysis investigated the risk of the other addictive disorder over a four-year period after the onset of GD and SUD, respectively, over age and sex. Among SUD patients, the risk of GD was greater among males compared to females, except for those aged 40–66 years developing GD three to four years after the onset of SUD where no sex differences were found. Among GD patients, although being male is a general risk factor for SUDs, we found this not to be consistent over different age groups or time periods. The risk of SUD was similar between males and females in the same year and one to two years after the onset of GD among those aged 18–39 years, whereas being female was a risk factor for SUD three to four years after the onset of GD. Similarly, being female was a risk factor one to four years after the onset of GD among GD patients aged 40–66 years. Previous findings suggest that a higher proportion of female problem gamblers are escape gamblers (e.g. gamble to alleviate depressed mood), whereas a higher proportion of male problem gamblers continue to gamble for economic reasons (e.g. chasing losses) [29]. Accordingly, it may be hypothesised that the higher proportion of female problem gamblers developing SUD a year or more after GD is linked to unsuccessful treatment of the gambling problem, leading to replacement of GD with SUD.

### Limitations and strengths

Some limitations of the present study should be noted. As the NPR was founded in 1997 and the PIN was introduced in 2008, it is likely that some patients were diagnosed with GD and SUD before 2008. However, the number of patients diagnosed with GD and SUD before 2008 is not known. Time between treatments was measured by calendar year. Therefore, the time interval between first registered incidence of GD and/or SUD may be considered imprecise. Furthermore, the study did not differentiate between different SUDs, such as alcohol, different drugs, tobacco and caffeine use disorder. Previous studies show that AUD and DUD are the most prevalent SUD disorders among GD patients [14]. The present study accounted for the association with age and sex, whereas homogeneity was assumed across other potentially important moderating factors such as health (e.g. disorder severity, number of treatments and medical use among the patients) and socio-demographic factors (e.g. income, family status, employment status, etc.). Not taking into account the effect of these potentially important factors may result in misleading interpretations of the homogeneity of those with SUD and GD. As such, future studies should investigate whether the prospective relationships investigated in the present study differ between groups with different general health vulnerabilities (e.g. suffering from another mental disorder), as well as accounting for socio-demographic factors associated with GD and SUD. Finally, the study population was restricted to those treated by public health authorities and private clinics contracted by public health authorities. Therefore, those treated by private treatment agencies not contracted by public Norwegian health authorities were not included.

In terms of strengths, the present study is one of very few studies worldwide that exclusively utilised health registry data. Therefore, memory and other reporting biases could not have influenced the findings. The present study also comprised the whole population of those diagnosed with SUD and GD registered within the NPR, covering a period of 10 years. Another very important asset is the prospective design, providing tentative indications about the directionality between SUD and GD.

## Conclusions

The present study examined the co-morbidity between GD and SUD using nationwide health registry data of a first-time incidence of GD and SUD from 2008 to 2017 in Norway. The co-morbidity between GD and SUD was low. However, the prevalence of the other addictive disorder appears to depend on the nature of the first disorder, where GD patients have a greater risk of subsequent SUD than vice versa. There are sex differences in the risk trajectories of the other addictive disorder over time between GD patients and SUD patients, indicating a need to replicate and examine the co-morbidity among GD and SUD over time.
